# Supplementary material for: Response of psychrophilic plant endosymbionts to experimental temperature increase
Source: R Soc Open Sci. 2020 Dec 2;7(12):201405. doi: 10.1098/rsos.201405 (PMC7813268; doi:10.1098/rsos.201405)
Supplement: Supplementary material [file rsos201405supp1.pdf]

SUPPLEMENTARY INFORMATION

Figure S1. Growth curves groups (G1, G2 and G3) based on ANOVA.

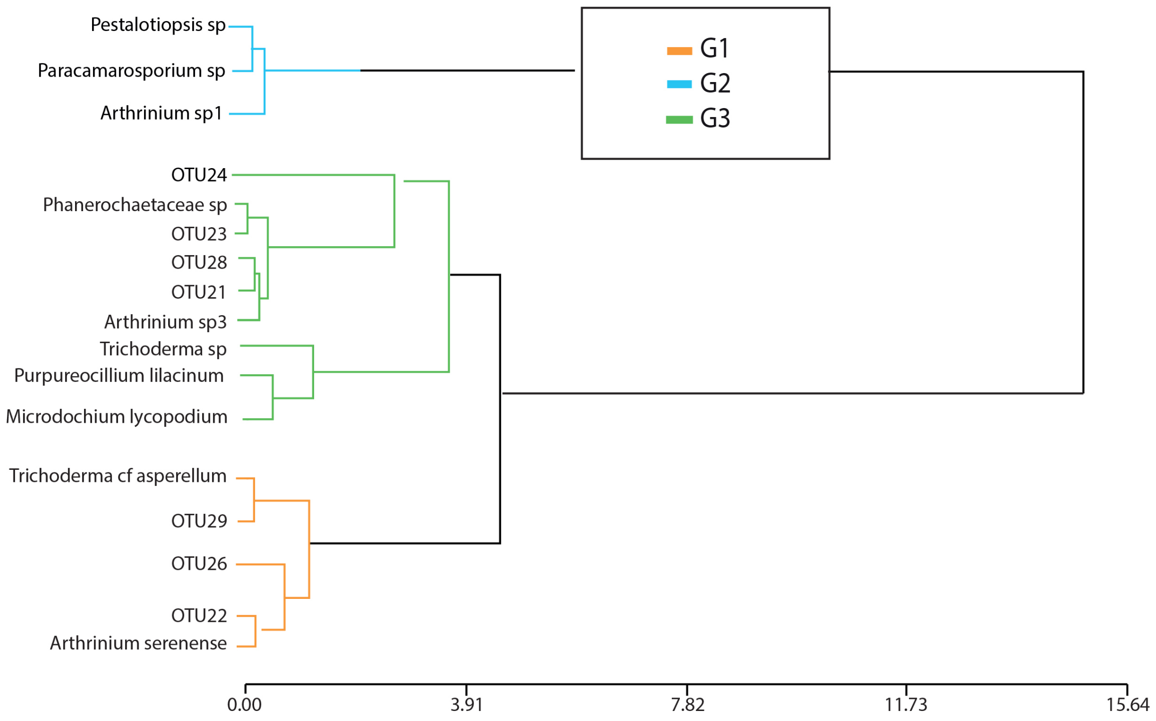

Figure S2. Average radial growth (mm) of the three groups of growth curves based on ANOVA.

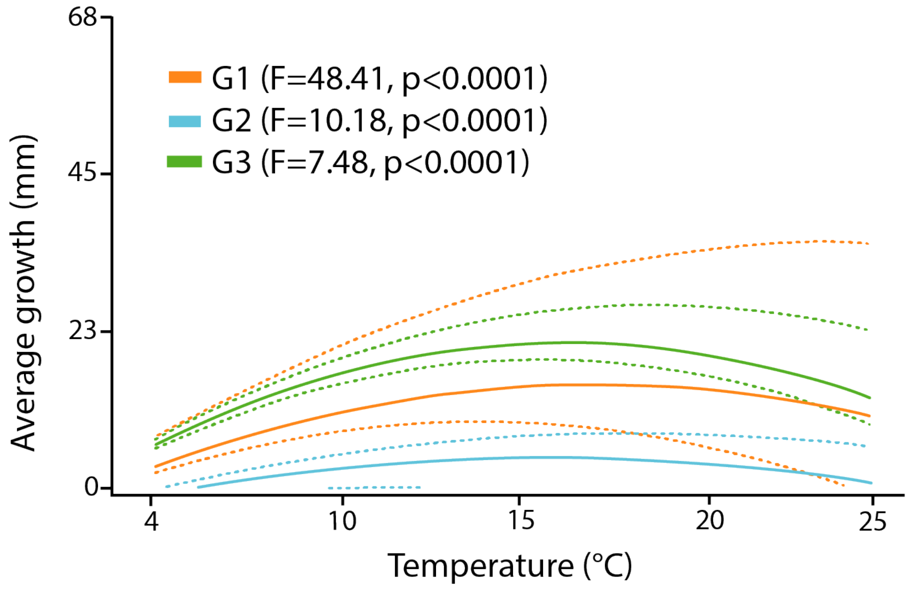

Figure S3. Initial growth curves, daily growth curves during the experiment of 5°C increase, temperature changes between day and night and final growth curves. All the growths measured in mm.

Initial growth curve	Experiment	Final growth curve

5°C increase

1

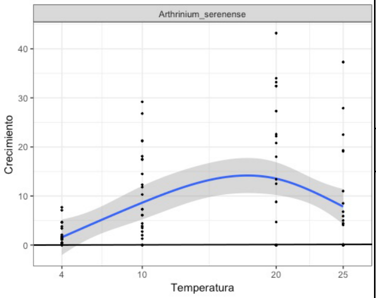

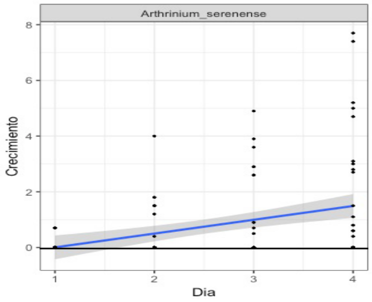

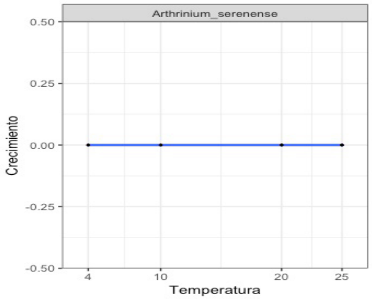

Day/Night Shift

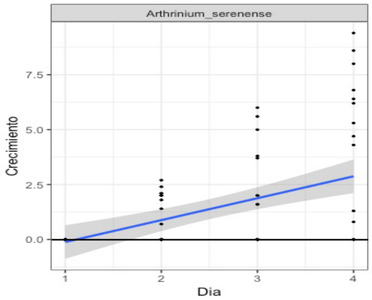

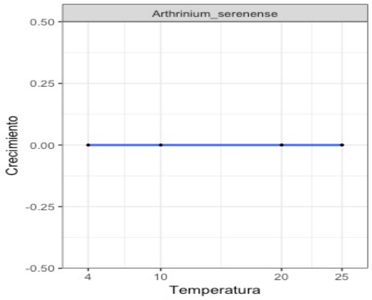

5°C increase

2

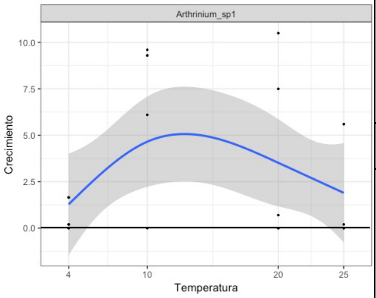

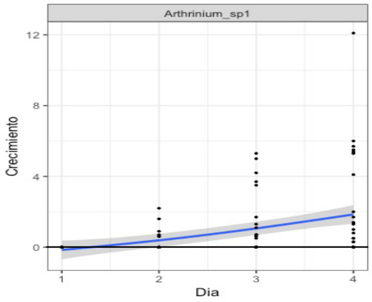

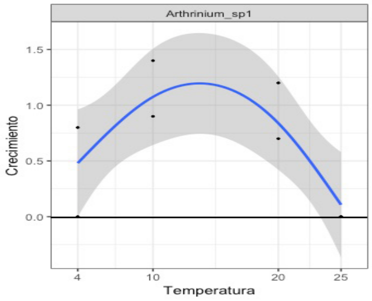

Day/Night shift

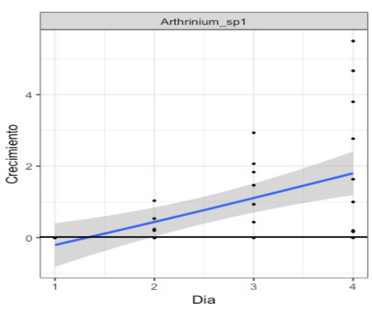

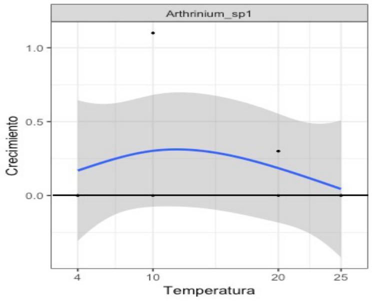

5°C increase

3

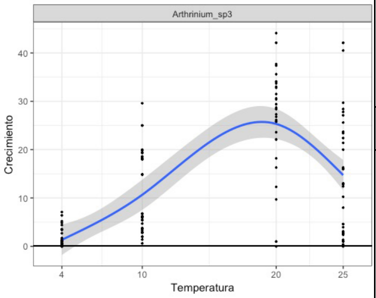

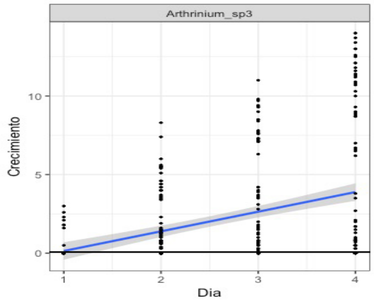

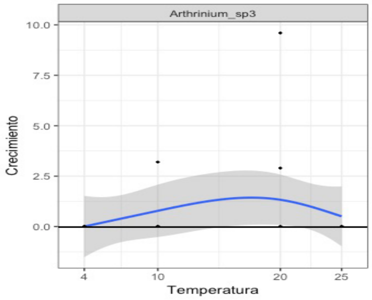

Day/Night shift

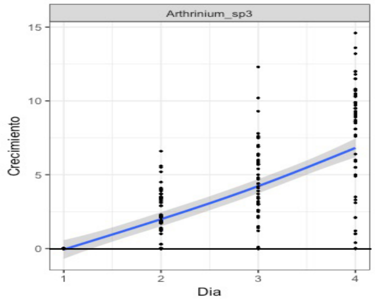

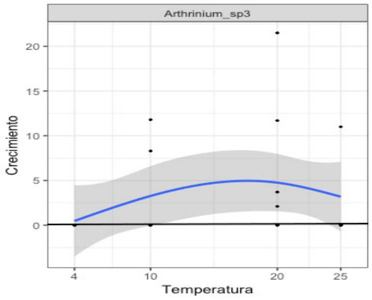

4

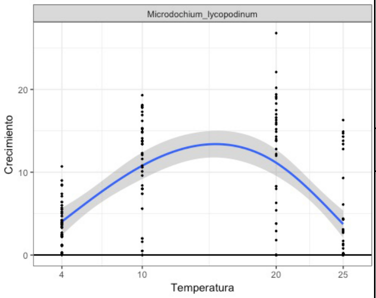

5°C increase

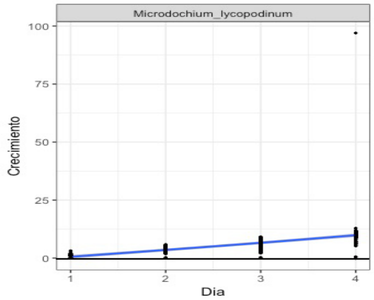

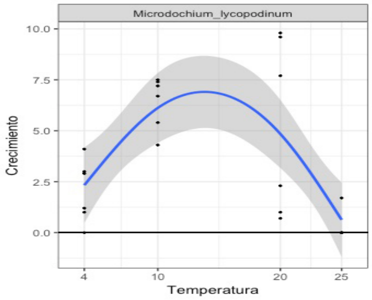

Day/Night shift

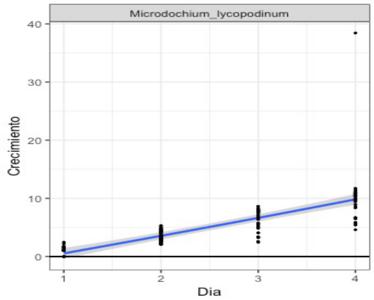

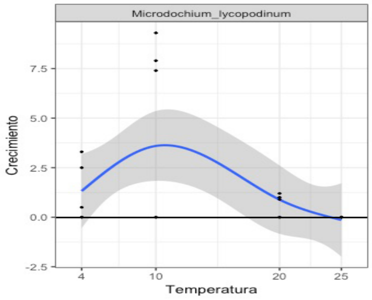

5

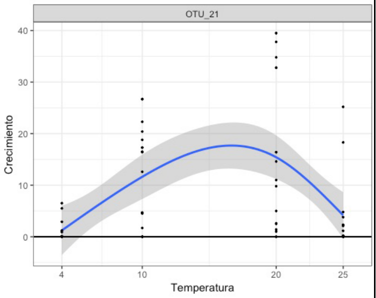

5°C increase

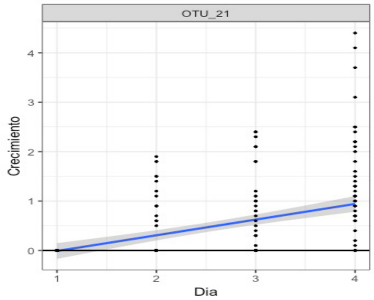

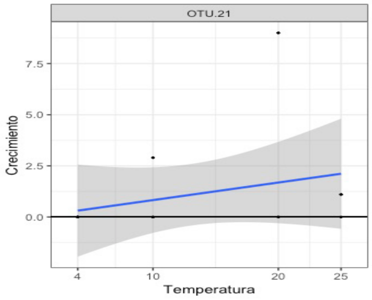

Day/Night shift

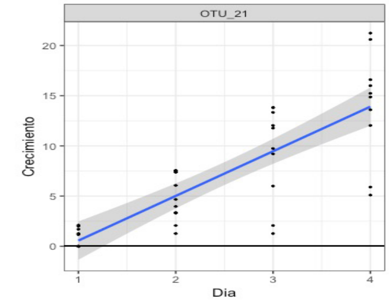

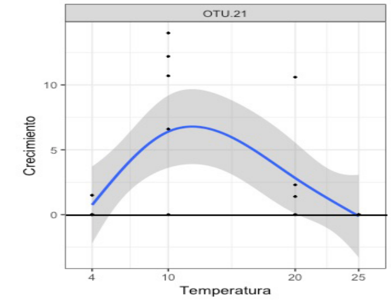

6

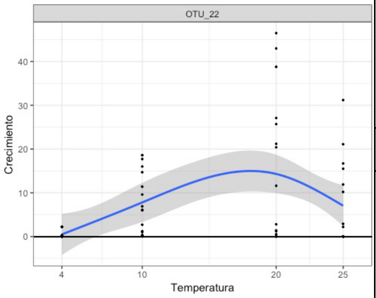

5°C increase

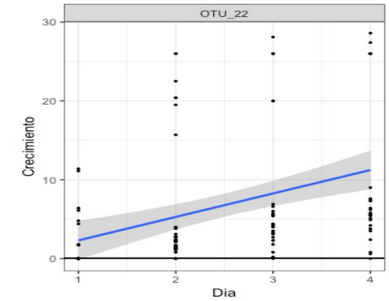

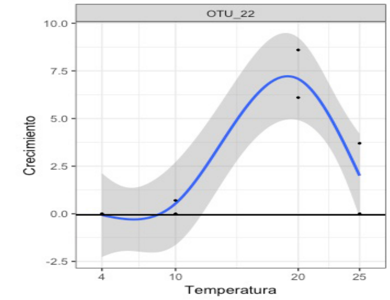

Day/Night shift

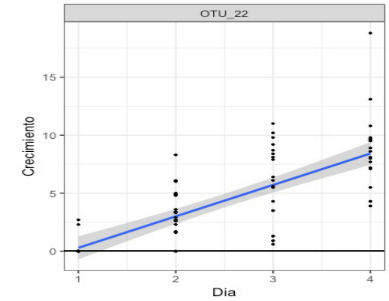

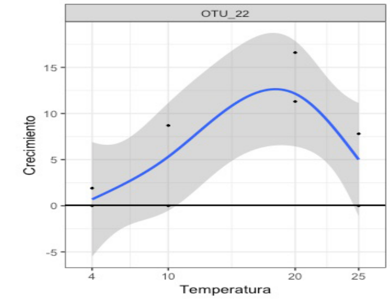

7

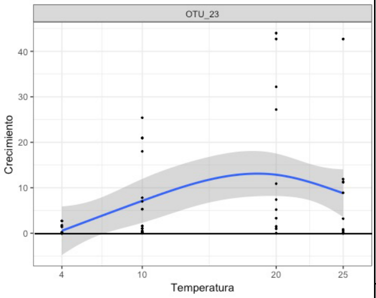

5°C increase

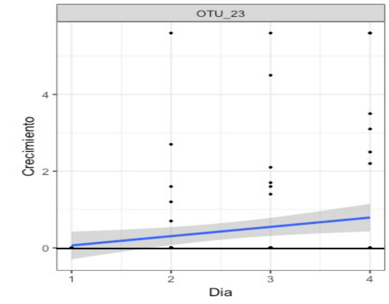

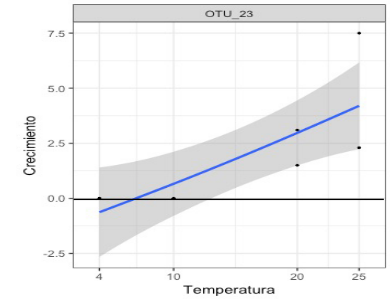

Day/Night shift

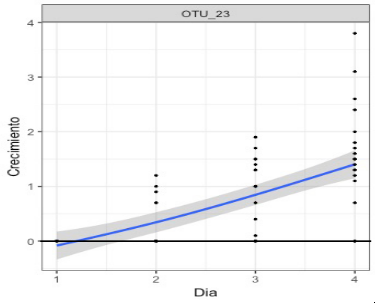

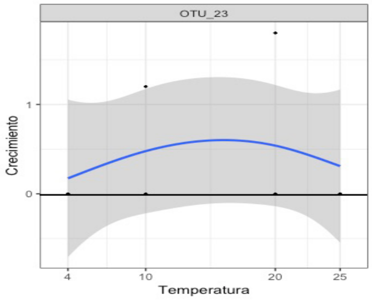

8

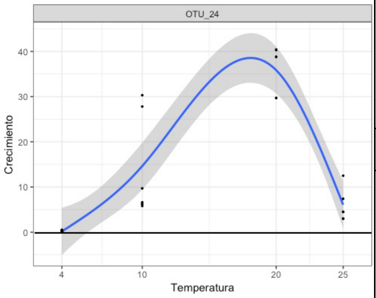

5°C increase

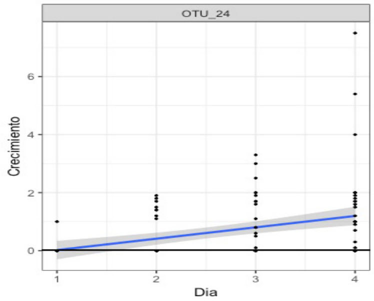

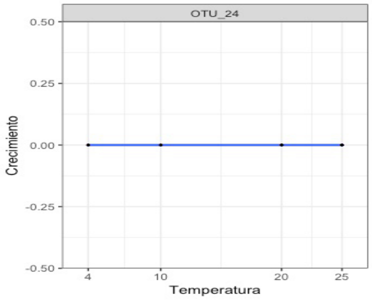

Day/Night shift

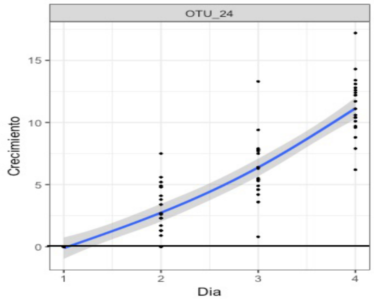

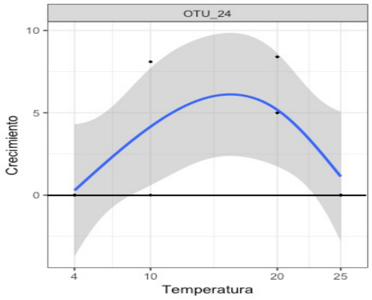

9

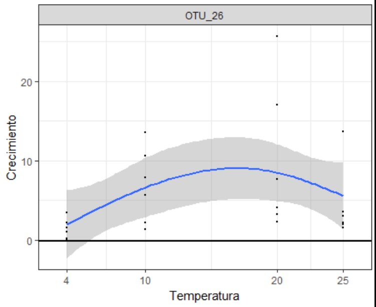

5°C increase

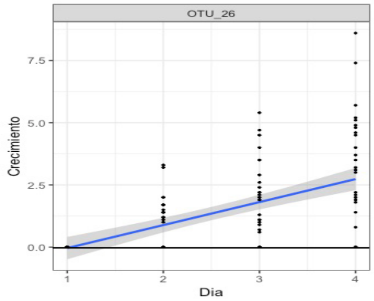

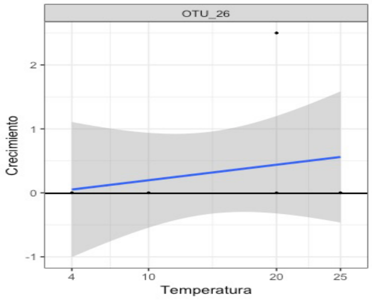

Day/Night shift

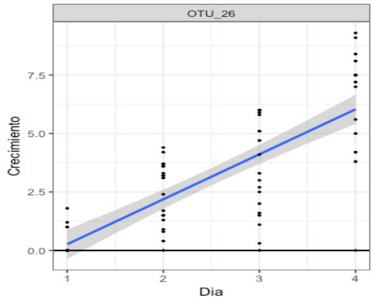

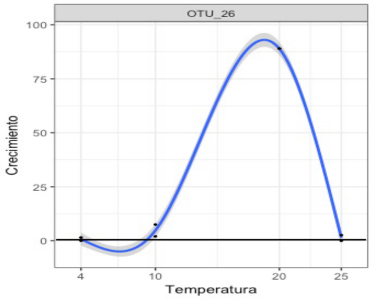

10

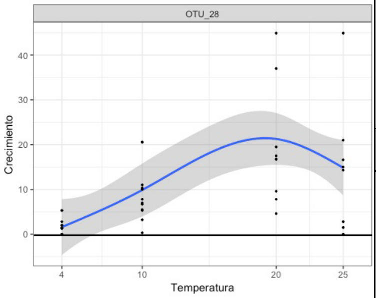

5°C increase

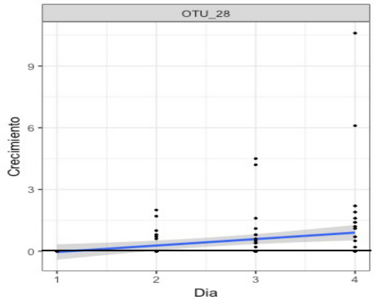

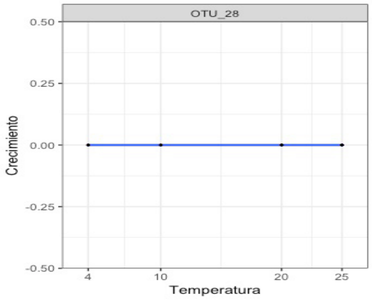

Day/Night shift

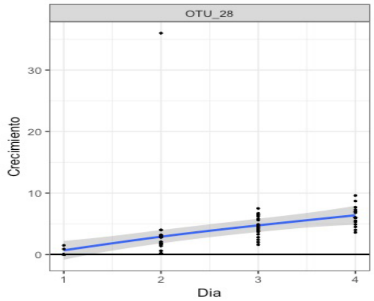

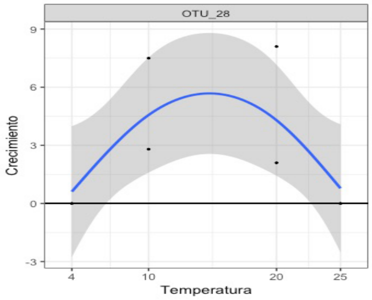

11

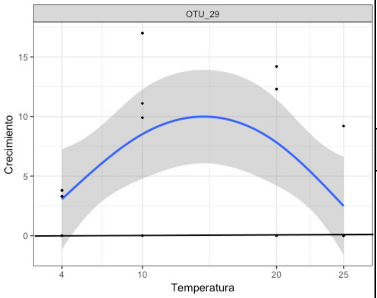

5°C increase

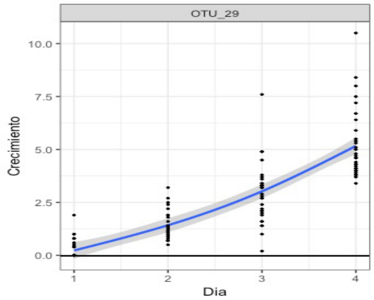

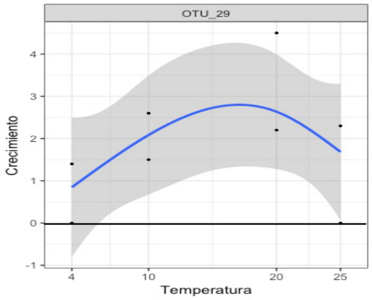

Day/Night shift

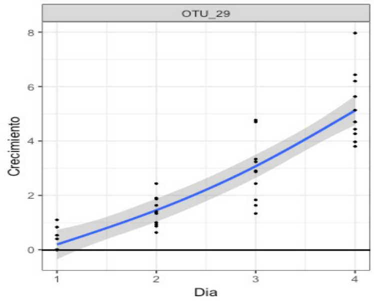

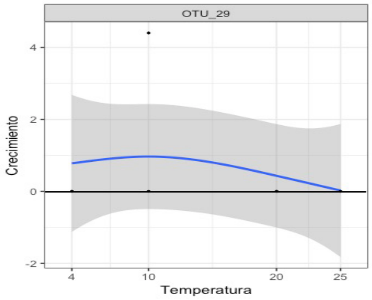

5°C increase

12

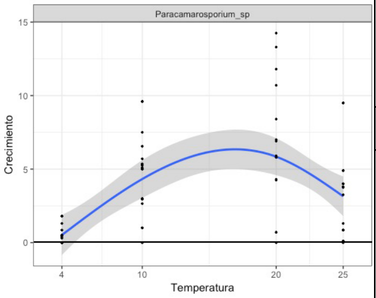

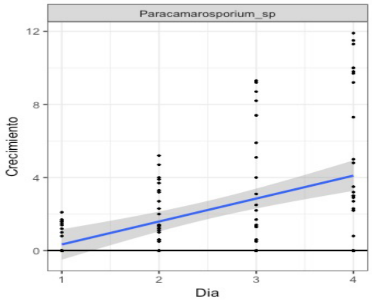

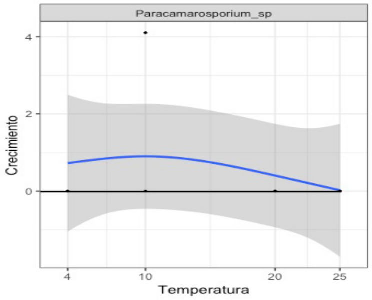

Day/Night shift

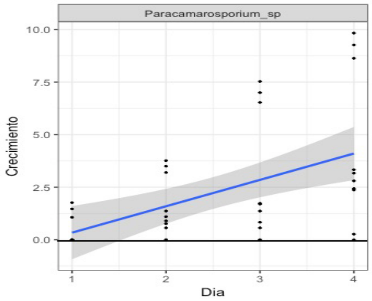

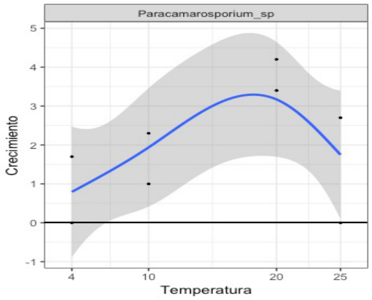

5°C increase

13

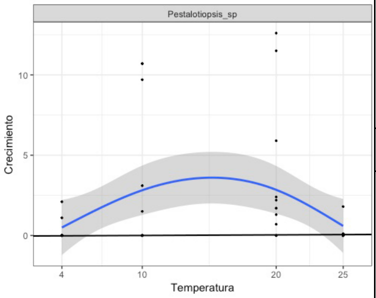

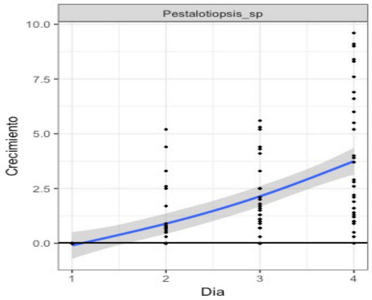

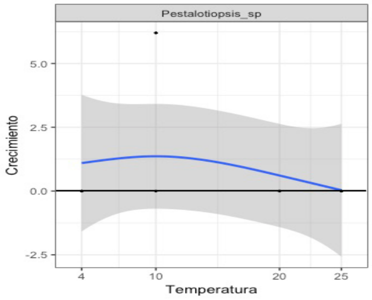

Day/Night shift

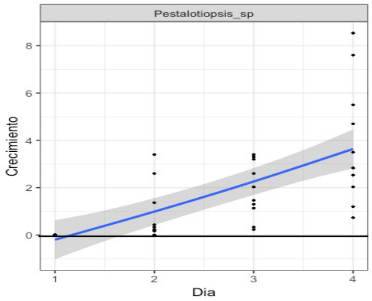

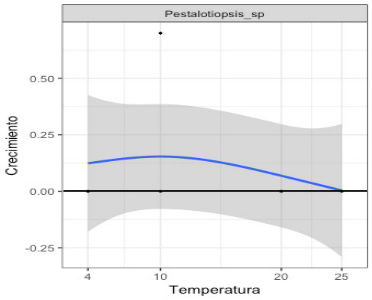

14

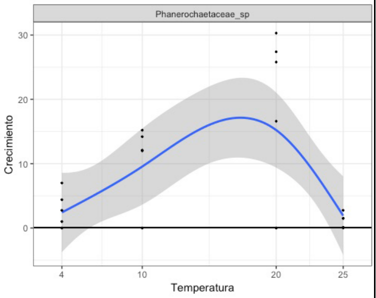

5°C increase

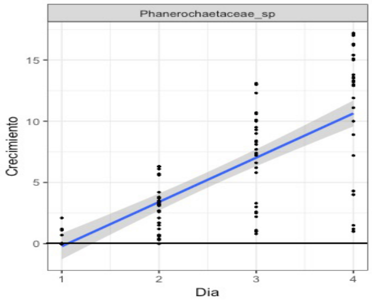

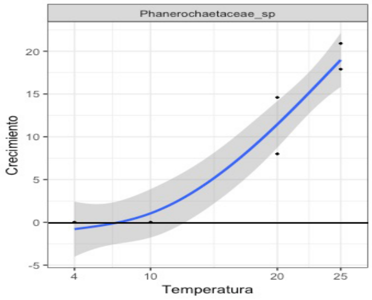

Day/Night shift

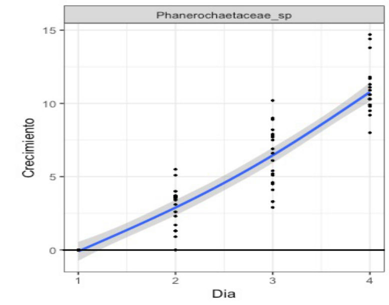

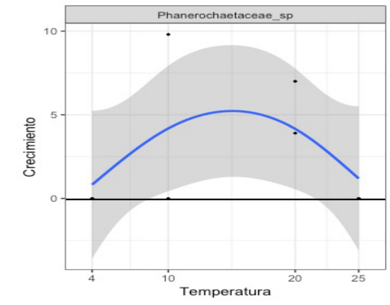

15

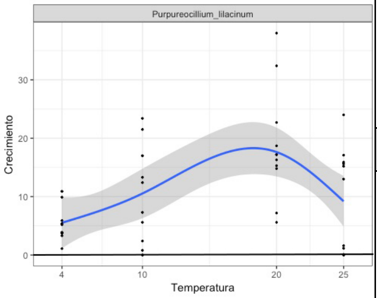

5°C increase

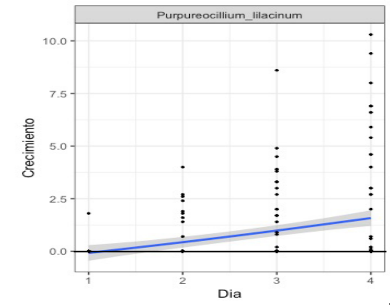

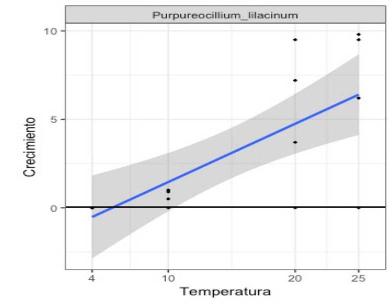

Day/Night shift

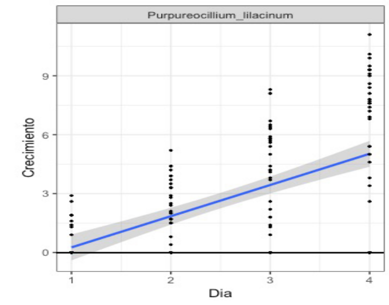

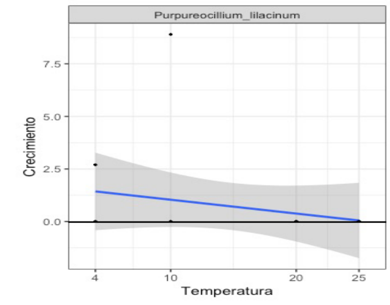

16

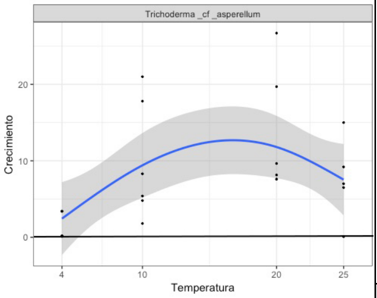

5°C increase

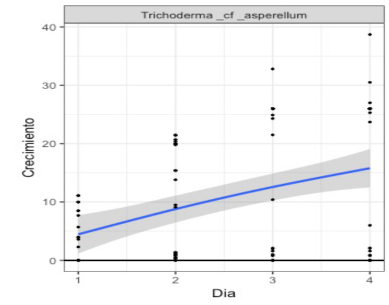

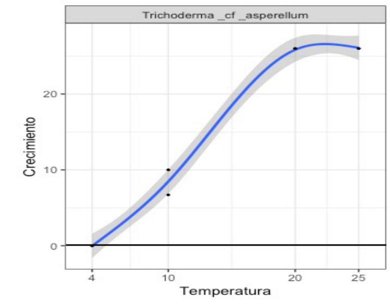

Day/Night shift

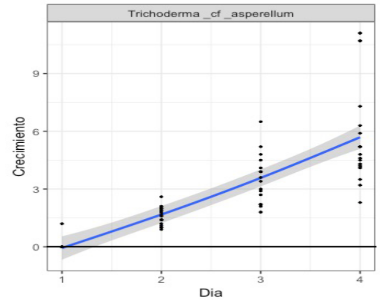

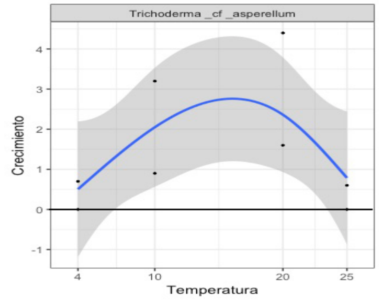

17

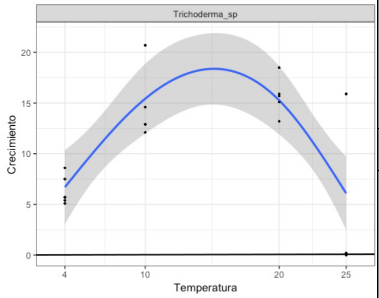

5°C increase

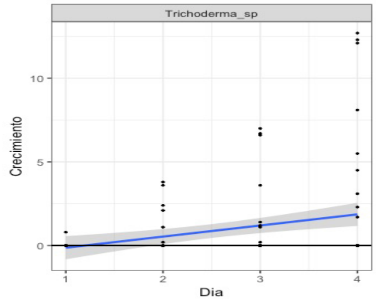

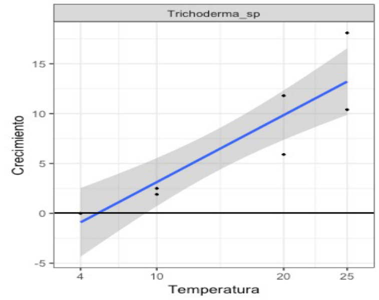

Day/Night shift

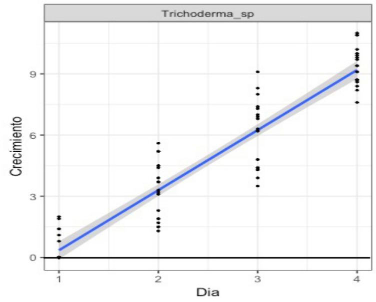

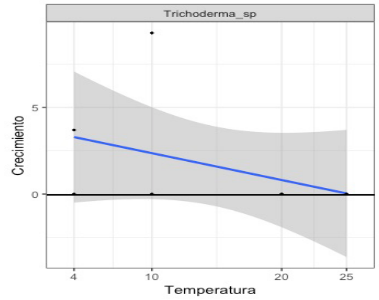

Figure S4. Location of the study areas: Cerro Buena Vista (3 491m) and Cerro Chirripó (3 819m). Adapted from: Danhy Estid Fuentes (2014). Source: Atlas de Costa Rica (2014).

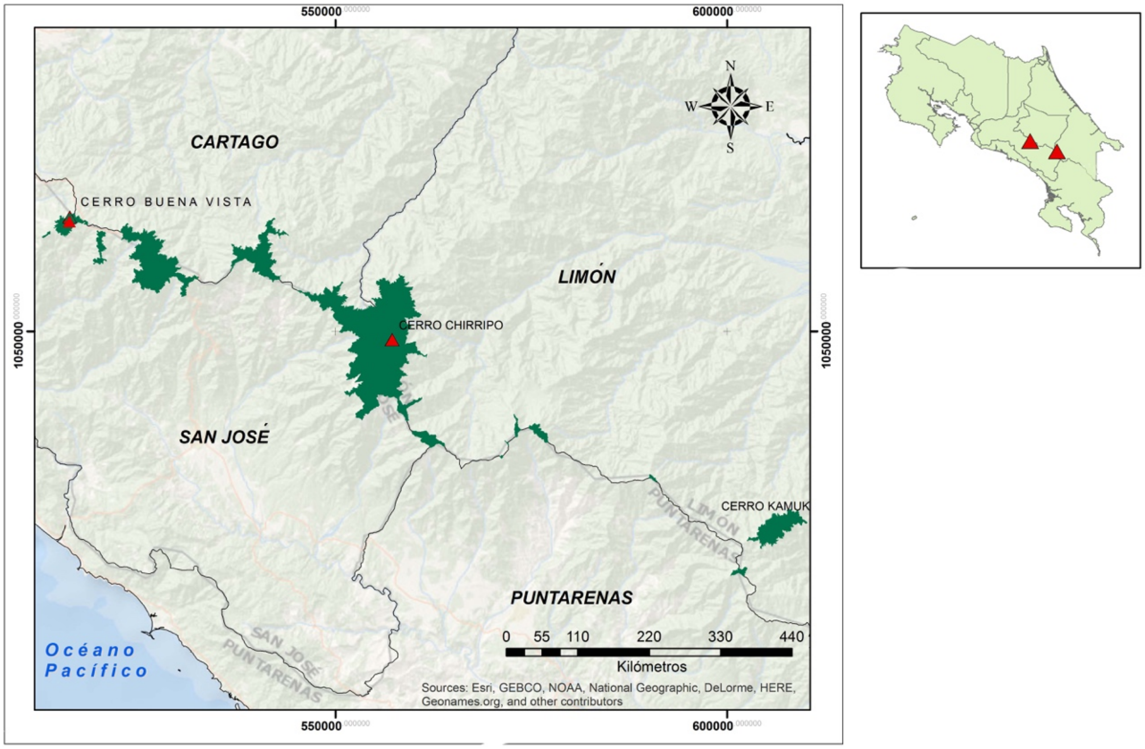

Supplementary Figure S5. Diagram of the experimental design of plots subdivided in Latin Square to evaluate, in the laboratory, the growth response of endophytic psychrophilic fungi associated to *Chusquea subtessellata* (Poaceae) to a range of temperatures.

Replicate	Chamber 1	Chamber 2	Chamber 3	Chamber 4
1	A	B	C	D
2	B	C	D	A
3	C	D	A	B
4	D	A	B	C

A = 4°C, B = 10°C, C=20°C, D=25°C

Figure S6. Diagram of the experiment of adaptation to increase in temperature.

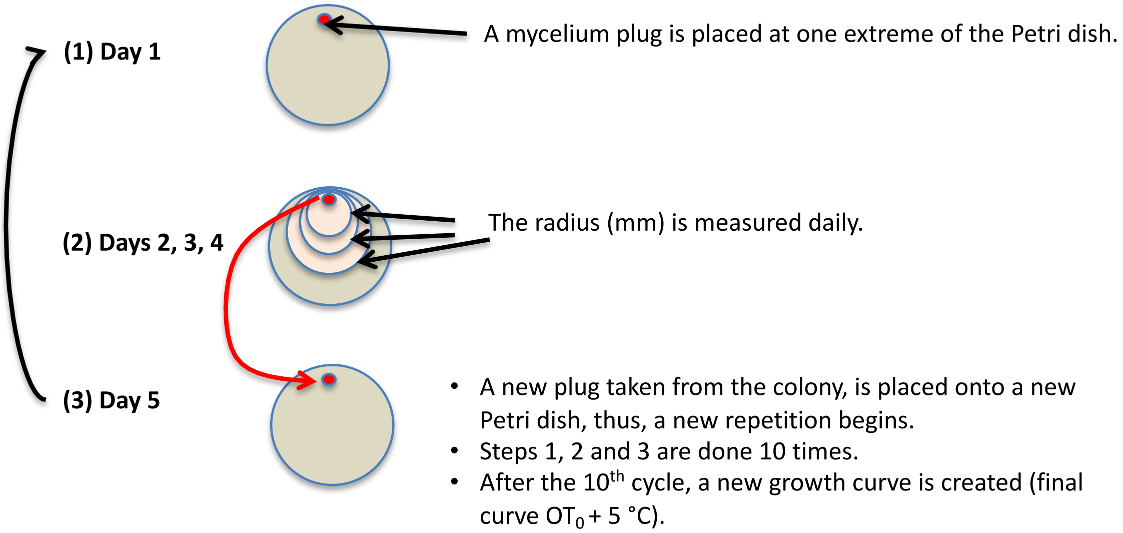

Supplementary Table S1. Results of BLAST search and fungal OTU identification. Information according to site and plant part is also presented.

Isolate#	Genbank accession #	Fungal OTU identification	Query cover %	% identity	Buena Vista			Chirripó		
					Leaf	Root	Stem	Leaf	Root	Stem
CS19	MT882143	*Arthrinium serenense**	100	99			X	X		X
CS20	MT882153		100	97						
CS27	MT882145		100	97						
CS10	MT882133	*Arthrinium* sp1	100	92				X		
CS14	MT882171	*Arthrinium* sp2	100	97					X	
CS11	MT882141	*Arthrinium* sp3**	78	94			X	X	X	
CS40	MT882197		76	94						
CS41	MT882129		76	94						
CS33	MT882127	*Aureobasidium pullulans*	99	98						X
CS32	MT882195	*Cladosporium* cf. *varians*	100	100				X		
CS7	MT882179	Unident. Cordycipitaceae	99	93				X		
CS17	MT882123	Unident. Helotiales	99	95					X	
CS5	MT882159	*Microdochium lycopodinum**	100	100	X			X	X	
CS13	MT882161		100	100						
CS15	MT882181		100	100						
CS24	MT882193		100	100						
CS25	MT882125		100	100						
CS51	MT882149		100	100						
CS36	MT882155	Unident. Ophiostomataceae	100	97					X	
CS37	MT882167		100	97						
CS6	MT882169	*OTU_21*	N/A	N/A	X					
CS46	MT882177	*OTU_22*	N/A	N/A			X			
CS44	MT882157	*OTU_23**	N/A	N/A	X			X		
CS12	MT882151									
CS38	MT882175	*OTU_24*	N/A	N/A		X				

CS30	MT882173	*OTU_25*	N/A	N/A				X		
CS16	MT882191	*OTU_26*	N/A	N/A						X
CS2	MT882131	*OTU_27*	N/A	N/A				X		
CS39	MT882187	*OTU_28*	N/A	N/A						X
CS26	MT882137	*OTU_29*	N/A	N/A					X	
CS31	MT882185	*Paracamarosporium* sp.	100	95					X	
CS8	MT882189	*Pestalotiopsis* sp.*	100<br>100	99<br>99	X			X		
CS35	MT882147	*Pezicula melanigena*	100	100					X	
CS23	MT882183	*Purpureocillium lilacinum*	100<br>100	100<br>100					X	X
CS50	MT882139	*Sterigmatomyces halophilus*	100	100		X				
CS29	MT882165	*Trichoderma* cf. *asperellum*	100	99					X	
CS18	MT882135	*Trichoderma* cf. *viridescens*	100	98					X	
CS21	MT882163	Unident. Xylariaceae	99	88				X		

*Matches in site and plant part ** Matches in site only
